# Autoimmune Glial Fibrillary Acidic Protein (GFAP) Astrocytopathy Presenting as Viral Encephalitis: A Case Report and Literature Review

**DOI:** 10.7759/cureus.98580

**Published:** 2025-12-06

**Authors:** Soorya Bavikeri Shivakumara Hegde, Janak Sureshbhai Nayak, Muna Salah, Jeeth Thomas, Brendan Davies

**Affiliations:** 1 Internal Medicine, University Hospitals of North Midlands NHS Trust, Stafford, GBR; 2 Acute Medicine, University Hospitals of North Midlands NHS Trust, Stafford, GBR; 3 Neurology, University Hospitals of North Midlands NHS Trust, Stafford, GBR

**Keywords:** autoimmune encephalitis, clinical case report, glial fibrillary acidic protein, immune-mediated neurological diseases, viral encephalitis mimic

## Abstract

Glial fibrillary acidic protein astrocytopathy is a rare autoimmune condition characterized by antibodies against glial fibrillary acidic protein in astrocytes of the central nervous system. It can present with features of myelitis or meningoencephalitis. Diagnosis is based on the presence of serum or cerebrospinal antibodies against glial fibrillary acidic protein. Most cases respond to immunosuppressive therapy in the form of steroids.

A 47-year-old woman with dizziness, tremors, and coryzal symptoms progressed over a period of a few weeks to develop clinical features of brainstem encephalitis. She was started on empirical antimicrobials and anti-convulsive therapy. Although initial MRI showed features of viral encephalitis, this was ruled out after CSF testing, and she was given high-dose methylprednisolone. She was admitted to the intensive care unit in view of her deteriorating level of consciousness. There was an excellent response to steroids, raising the probability of an autoimmune process, and she was extensively investigated over the next two weeks by a multidisciplinary team. Glial fibrillary acidic protein immunoglobulin G antibodies were detected in her serum and cerebrospinal fluid. She was started on a dose-tapering regimen of steroid and anti-convulsant medication along with rehabilitation therapy. She continues to make good progress. In conclusion, autoimmune glial fibrillary acidic protein astrocytopathy is a rare clinical entity that can be a diagnostic challenge. It’s essential to consider this in the differentials of cases presenting with infectious meningoencephalitis to institute early immunosuppressive therapy and improve clinical outcome.

## Introduction

Glial fibrillary acidic protein (GFAP) astrocytopathy was first described in 2016 [1]. It is a rare autoimmune condition that can involve various parts of the central nervous system (CNS), like the subcortical white matter, brainstem, medulla, hypothalamus, basal ganglia, or spinal cord. An incidence of 0.03 per 100,000 person-years and a prevalence of 0.6 per 100,000 people have been reported in a previous study [2]. GFAP astrocytopathy has been reported in both adults and children. It is more commonly seen in middle-aged adults, with a slight male preponderance [3].

Inflammation from certain immune cells (lymphocytes and granulocytes), support cells (astrocytes), and the complement system has been implicated in the pathogenesis [4]. This results in features of meningitis, encephalitis, or myelitis, like fever, headache, abnormal vision, altered consciousness, seizures, movement disorders, hyperreflexia, and ophthalmoplegia [5]. Due to its similarity to infections, such as viral encephalitis, it is often misdiagnosed or initially mistaken for an infectious process. The relationship between viral infections and GFAP astrocytopathy is still an area of active research. A recent meta‐analytical study revealed that 45% of patients had a viral prodrome, primarily upper respiratory or gastrointestinal infections, with some rarer cases involving HIV and bacterial infections [3]. The time between the appearance of viral symptoms and the onset of neurological manifestations is usually brief, typically occurring within weeks to months [3]. Although it is part of the autoimmune encephalitis spectrum, it is distinct in its pathophysiology and clinical presentation, and is characterized by specific immune reactions involving glial fibrillary acidic protein immunoglobulin G antibodies (GFAP-IgG) in cerebrospinal fluid (CSF) [6]. Magnetic resonance imaging can show typical findings, such as periventricular radial and linear contrast enhancement [7,8], and treatment involves steroids or intravenous immunoglobulin therapy [9].

We present a case of a middle-aged woman where the diagnosis was protracted due to the clinico-radiological resemblance to infectious encephalitis. We also emphasize the importance of early consideration of autoimmune mechanisms in the diagnostic process and the prompt initiation of appropriate therapy to improve patient outcomes.

## Case presentation

Initial presentation (admission days one and two)

A 47-year-old Caucasian woman with a history of type 2 diabetes mellitus was initially seen in the general practice for dizziness, for which she was given prochlorperazine 5 mg tablets (to take as required). When her symptoms worsened, she presented to the Accident and Emergency (A&E) department. Neurological examination was unremarkable, and blood tests were as shown in Table 1. She was given cyclizine 50 mg tablets for vestibular neuritis and discharged. She then developed worsening dizziness, tinnitus, poor balance, and new-onset tremors with no other neurological symptoms. This was put down to the side effects of cyclizine therapy and treated with betahistine 16 mg tablets. She was re-admitted to A/E with worsening dizziness, tremors, loss of balance, agitation, and hallucinations. The patient was unable to stand due to ataxia and tremor, with an initial Glasgow Coma Scale (GCS) of 15/15. Blood tests showed raised infection markers and acute kidney injury (Table 1). A panel of tests done as part of a full septic screen, to rule out other causes of infection, were unremarkable. Following specialist input obtained from the neurology and infectious diseases team, she was treated along the lines of meningoencephalitis with 2 g of intravenous (IV) ceftriaxone twice a day, 10 mg/kg of IV acyclovir three times a day, and 500 mg of IV metronidazole given three times a day. These antimicrobials were given for a total of 14 days. She was also given 3 g of intravenous levetiracetam and 2 g of IV sodium valproate as a loading dose, followed by 1.5 g of levetiracetam twice a day and 600 mg of sodium valproate three times a day intravenously for maintenance to cover non-convulsive status epilepticus. She was given sedation for agitation and started on variable rate insulin infusion due to the risk of impending diabetic ketoacidosis (DKA). GCS had now dropped to 3, and computed tomography (CT) of the brain showed no intracranial abnormalities to explain the drop in her GCS.

**Table 1:** The results of initial blood tests. A/E: accidents and emergency; eGFR: estimated glomerular filtration rate

Parameter	First admission to A/E	Second admission to A/E	Reference range
Sodium	131 mmol/L	137 mmol/L	133-146 mmol/L
Potassium	3.7 mmol/L	3.8 mmol/L	3.5-5.3 mmol/L
Urea	7.6 mmol/L	14.9 mmol/L	2.5-7.8 mmol/L
Creatinine	49 mmol/L	96 mmol/L	45-84 mmol/L
eGFR	>90 mL/min/1.73 m²	6 mL/min/1.73 m²	>90 mL/min/1.73 m²
Magnesium	-	0.56 mmol/L	0.70-1.00 mmol/L
Inorganic phosphate	-	0.84 mmol/L	0.80-1.50 mmol/L
Ionized calcium	-	1.22 mmol/L	1.16-1.31 mmol/L
C-reactive protein (CRP)	-	8 mg/L	<4 mg/L
Albumin	-	40 g/L	35-50 g/L
Alkaline phosphatase	-	63 IU/L	30-130 IU/L
Alanine aminotransferase	-	32 IU/L	0-34 IU/L
Serum bilirubin (total)	-	26 mmol/L	0-21 mmol/L
Serum ammonia	-	27 mmol/L	0-40 mmol/L
Hemoglobin	157 g/L	157 g/L	115-165 g/L
Red blood cell count	5.80x10^12^/L	5.84x10^12^/L	3.80-5.80x10^12^/L
White cell count	12.50x10^9^/L	24.60x10^9^/L	4-11x10^9^/L
Lymphocytes	1.18x10^9^/L	2.14x10^9^/L	1.5-4.0x10^9^/L
Monocytes	0.73x10^9^/L	0.76x10^9^/L	0.2-0.8x10^9^/L
Neutrophils	10.31x10^9^/L	21.43x10^9^/L	2.0-7.5x10^9^/L
Platelets	350x10^9^/L	512x10^9^/L	150-450x10^9^/L

ICU course (admission days three to 17)

**Figure 1:**
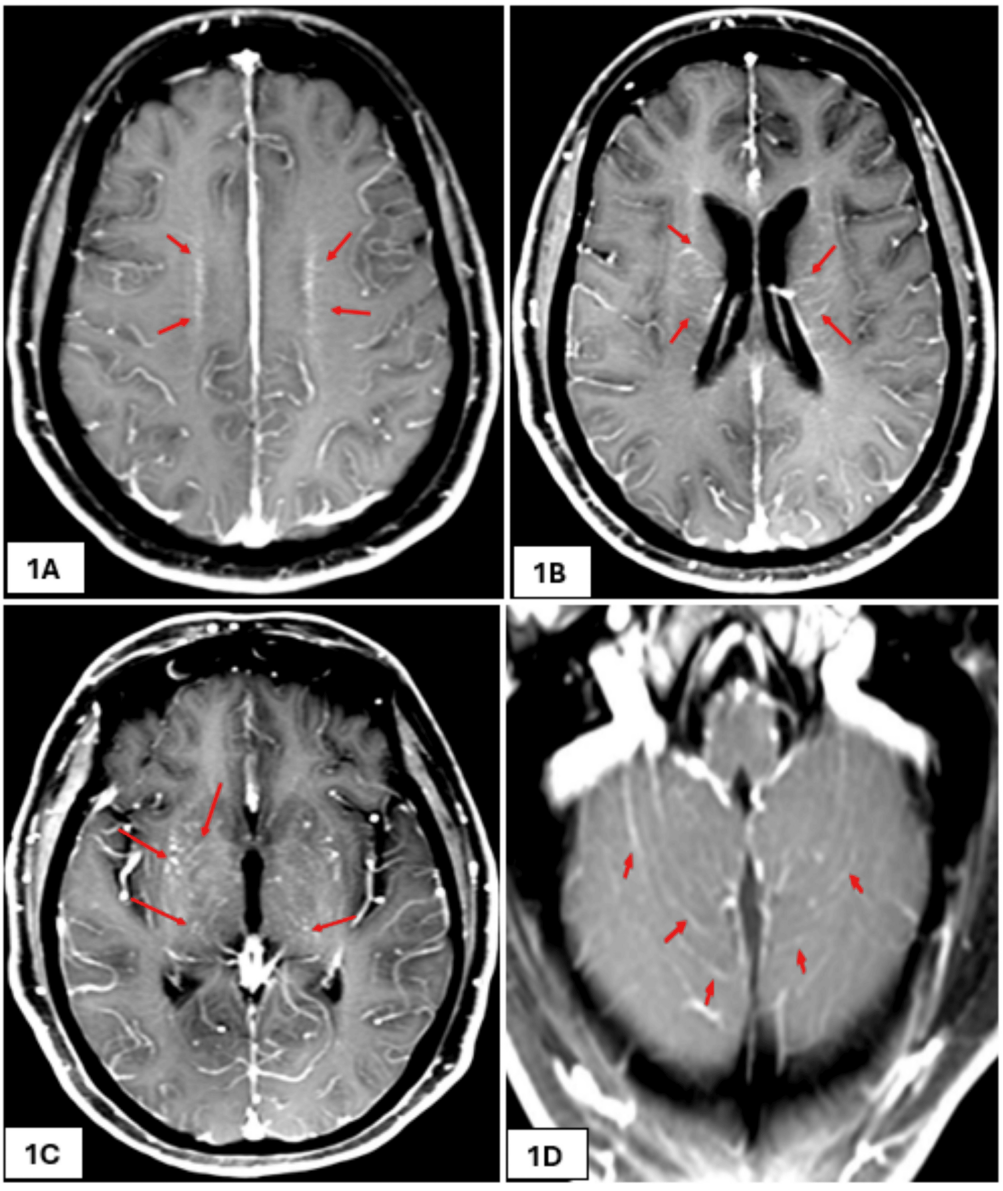
First MRI scan of brain (FAT-SAT axial images). Post-contrast T1 fat saturation (FAT-SAT) axial images showing bilateral symmetrical increased perivascular enhancement along the medullary veins - best appreciated at corona radiata level (long arrows in 1A) as well as basal ganglia and midbrain regions (long arrows in 1B and 1C) together with diffuse leptomeningeal enhancement extending to the scanned upper cervical cord (short arrows in 1D).

**Figure 2:**
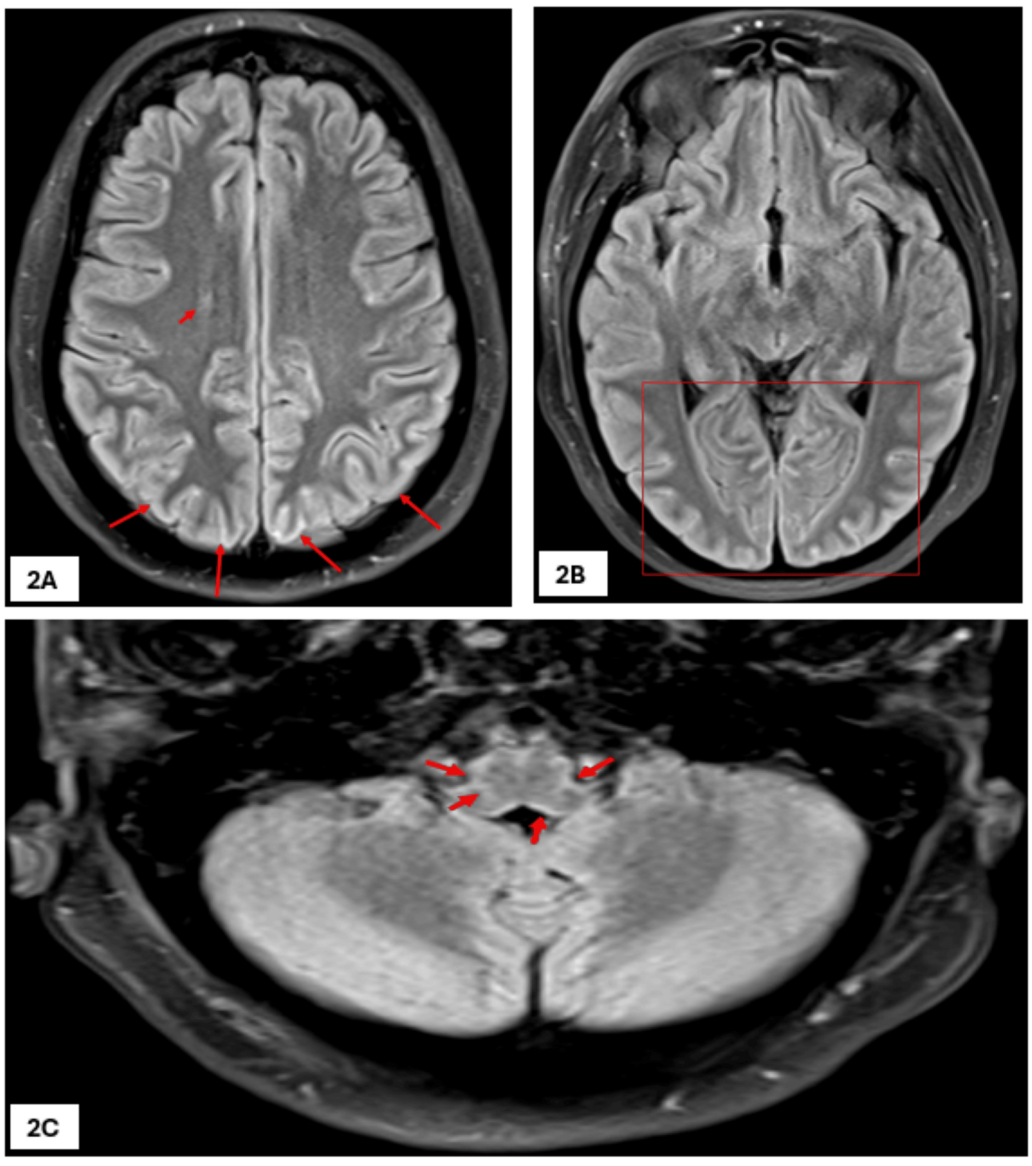
First MRI scan of brain (FLAIR images). Two-dimensional axial fluid-attenuated inversion recovery (FLAIR) images showing right frontal deep periventricular focus of FLAIR signal elevation (short arrow in 2A) with subtle cortical swelling and elevated FLAIR signal seen along high parietal (long arrows in 2A), posterior temporal and occipital polar regions (rectangle in 2B), and medulla oblongata (short arrows in 2C).

On initial assessment by the intensive care unit (ICU) team, her GCS had improved to 9/15. She was maintaining her airway and following commands. On further review, there was no improvement in her GCS, and she was noted to have clonus of the upper limbs. She was admitted to the ICU after intubation under sedation. CT venogram of the cerebral veins showed no evidence of cerebral sinus venous thrombosis, and magnetic resonance imaging (MRI) showed diffuse perivascular leptomeningeal enhancement in a posterior circulation distribution with minor parenchymal swelling, as shown in Figures 1A-1D, 2A-2C, which was suggestive of post-viral, perhaps post-COVID, encephalitis, as per the neuroradiologist. Once infective causes were included, she was started on 1 g methyl prednisolone per day intravenously for five days. A dramatic improvement was noted in her clinical status within two days of starting IV steroids. This was followed by a weaning dose of oral prednisolone tablets, which was started at 1 mg/kg body weight and reduced at a rate of 5 mg/week as per the advice from the neurologist. An electroencephalogram (EEG) done at this point showed a burst suppression pattern with no clear intermixed epileptic discharges.

Over the next two weeks in the ICU, she was managed by a multidisciplinary team consisting of a neurologist, a microbiologist, a rheumatologist, and an infectious diseases specialist. Clinically, she had convergent strabismus, head version to the left, initial anisocoria, involuntary myoclonus, asymmetric action tremors evolving into visual hallucinations, rhythmic hyperkinetic movements of her left shoulder, and bilaterally positive Hoffman’s sign. This was clinically suspected to indicate subacute brainstem encephalitis. Sodium valproate was now weaned down at the rate of 300 mg per day and then stopped. An extensive set of investigations was conducted to identify the cause of her presentation, with the results presented in Table 2. The results of the first lumbar puncture were unremarkable and are presented in Table 3.

**Table 2:** Results of further blood tests.

Parameters	Values	Reference range
Erythrocyte sedimentation rate (ESR)	7 mm/h	2-16 mm/h
Thyroid-stimulating hormone (TSH)	0.29 mIU/L	0.38-5.33 mIU/L
Triiodothyronine (T3)	3.8 pmol/L	3.5-6.5 pmol/L
Tetraiodothyronine (T4)	18.6 pmol/L	11.5-22.7 pmol/L
Thyroid peroxidase (TPO) antibody	Negative	Negative
Respiratory pathogens polymerase chain reaction (influenza A, influenza B, COVID-19, and human respiratory syncytial virus)	Not detected	-
Procalcitonin	0.15 µg/L	<0.05 µg/L
Legionella and Pneumococcal urinary antigens	Not detected	-
Pneumococcal and Meningococcal polymerase chain reaction (PCR)	Negative	-
Meningococcal group B polymerase chain reaction (PCR)	Negative	-
Multi-resistant Gram-negative bacterial culture screen	Not isolated	-
Blood cultures	Staphylococcus hominis	-
Sputum culture	No growth	-
General bacteriology swab culture (ear swab)	No growth	-
Tuberculosis microscopy (direct stain for acid/alcohol fast bacteria)	Not seen	-
Ebstein-Barr virus nuclear antigen antibody	IgG positive	-
Parvovirus B19 serology	IgG positive	-
Cytomegalovirus polymerase chain reaction (PCR)	Not detected	<500 IU/mL
BK virus polymerase chain reaction (PCR)	Not detected	-
JC virus polymerase chain reaction (PCR)	Not detected	-
*Borrelia burgdorferi* IgM and IgG antibodies	Negative	-
Brucella IgM/IgG antibody	<1:20	<1:20
Anti-extractable nuclear antigen antibodies (includes Ro, La, Smith, RNP, Jo1, and Scl70)	Negative	-
Anti-nuclear antibodies	Negative	-
Anti-myeloperoxidase antibodies	<0.7 U	<3.5 U
Anti-proteinase 3 antibodies	<0.3 U	<2 U
Paraneoplastic antibodies	Negative	-
Phospholipase A2 receptor antibody	<3.0 RU/mL	0-13 RU/mL
Cardiolipin immunoglobulin G antibody	0.7 GPL/mL	<10 GPL/mL
Glutamic acid decarboxylase (GAD) antibodies	<5.0 IU/mL	0-10 IU/mL
Myelin oligodendrocyte glycoprotein (MOG) antibodies	Negative	-
Aquaporin-4 antibodies	Negative	-
Acetylcholine receptor antibody	Negative	-
Muscle-specific tyrosine kinase IgG antibody	Negative	-
Herpes simplex virus 1 and 2 nucleic acid	Not detected	-
Varicella zoster virus nucleic acid	Not detected	-
HIV-1 and HIV 2 antigens and antibodies	Not detected	-
Treponema pallidum antibody screen	Not detected	-
Ganglioside antibodies (IgM and IgG) against GM1, GM2, GM3, GM4, GD1a, GD1b, GD2, GD3, GT1a, GT1b, GQ1	<1:500 (negative)	<1:500
Alpha fetoprotein	<1 kU/L	0-10 kU/L
Angiotensin converting enzyme (ACE)	28.2 U/L	13.3-63.9 U/L
Schirmer’s test (for Sjogren’s syndrome)	Right eye: 21 mm, left eye: 24 mm	≥10 mm/5 min
Anti-N-methyl-D-aspartate receptor	Negative	-
Alpha-amino-3-hydroxy-5-methyl-4-isoxazolepropionic acid receptor 1 and 2 antibody	Negative	-
Dipeptidyl-peptidase-like protein-6 antibodies	Negative	-
Contactin-associated protein 2	Negative	-
Leucine-rich glioma-inactivated protein 1	Negative	-
Gamma-aminobutyric acid receptor type B1	Negative	-
Glial fibrillary acidic protein immunoglobulin G (GFAP IgG)	Positive	-
Purkinje cell cytoplasmic antibody type 1 (PCA-1/Yo)	Negative	-
Anti-neuronal nuclear antibody type 1 (ANNA-1/Hu)	Negative	-
Anti-neuronal nuclear antibody type 2 (ANNA-2/Ri)	Negative	-
Ma antibody	Negative	-
Collapsin response-mediator protein-5/CV2 (CRMP5) antibody	Negative	-
Amphiphysin antibody	Negative	-
Anti-Sry-like high-mobility group box 1 (SOX1) antibody	Negative	-
Glutamic acid decarboxylase (GAD) antibody	Negative	-
Zinc-finger protein of the cerebellum 4 (Zic4) antibody	Negative	-
Delta/notch-like epidermal growth factor-related receptor (DNER) antibody	Negative	-

**Table 3:** Results of cerebrospinal fluid testing. *Included testing for the following organisms: *Escherichia coli *K1, *Haemophilus influenzae*, *Listeria monocytogenes*, *Neisseria meningitidis*, *Streptococcus agalactiae*, *Streptococcus pneumoniae*, Cytomegalovirus, Enterovirus, Herpes simplex virus 1, Herpes simplex virus 2, Human herpesvirus 6, Human parechovirus, Varicella zoster virus, and *Cryptococcus neoformans/gattii*.

Parameter	Values (1st sample)	Values (2nd sample)	Reference range
Total protein	1.44 g/L	0.51 g/L	0.15-0.45 g/L
Glucose	-	3.8 mmol/L	2.5-5.5 mmol/L
Red blood cells	88×10⁶/L	27×10⁶/L	×10⁶/L
White blood cells	359×10⁶/L	29×10⁶/L	0-5×10⁶/L
Polymorphs	0%	5%	-
Mononuclear cells	100%	95%	-
Organisms	No organisms seen	No organisms seen	-
Glial fibrillary acidic protein immunoglobulin G (GFAP IgG)	Not tested	Positive	-
Culture	No growth	No growth	-
ME film array*	None detected	Not tested	-
Anti-N-methyl-D-aspartate receptor	Negative	-	-
Alpha-amino-3-hydroxy-5-methyl-4-isoxazolepropionic acid receptor 1 and 2 antibody	Negative	-	-
Dipeptidyl-peptidase-like protein-6 antibodies	Negative	-	-
Contactin-associated protein 2	Negative	-	-
Leucine-rich glioma inactivated protein 1	Negative	-	-
Gamma-aminobutyric acid receptor type B1	Negative	-	-

**Figure 3:**
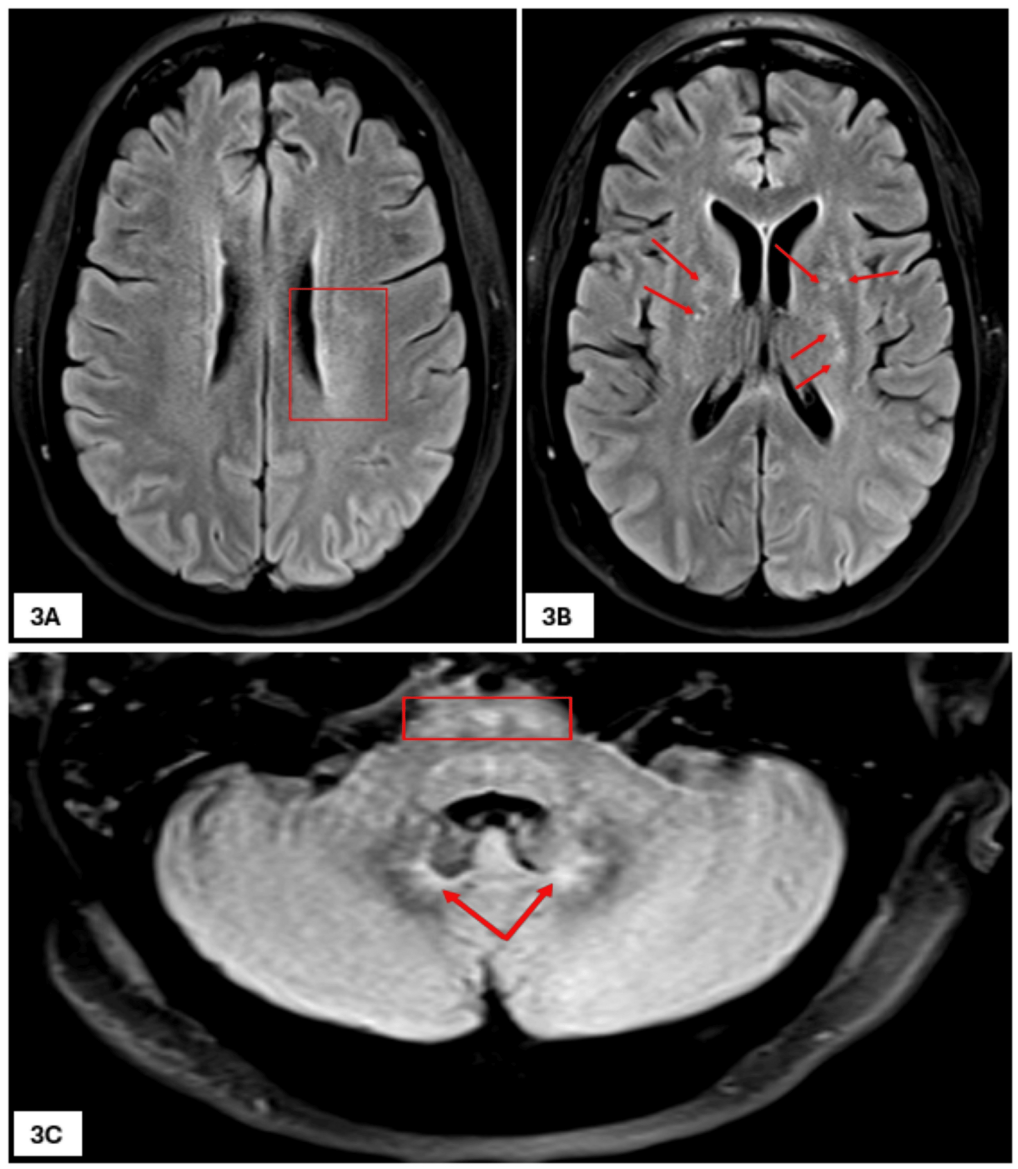
Second MRI scan of the brain. Two-dimensional axial FLAIR images showing progressive changes - bilateral deep periventricular white matter with indistinct areas of FLAIR signal alteration (rectangle in 3A); basal ganglia foci of elevated FLAIR signal (arrows in 3B); indistinct foci in the pontine tegmentum (rectangle in 3C); and the ventral aspects of both dentate nuclei (arrows in 3C). FLAIR: fluid-attenuated inversion recovery

Two further EEGs performed within a span of seven days did not show any evidence of epileptiform abnormalities. A second MRI of the brain showed progressive development of bilateral deep periventricular white matter and basal ganglia foci of elevated fluid-attenuated inversion recovery (FLAIR) signal (Figures 3A-3C). MRI of the spine, however, did not show any significant findings. CT of thorax, abdomen, and pelvis showed bilateral incidental pulmonary embolism during this stay, for which she was initially started on treatment dose of dalteparin given subcutaneously once a day and later changed to apixaban 10 mg tablets taken twice a day for seven days, followed by 5 mg tablets twice a day for maintenance.

Recovery (admission days 21-48)

**Figure 4:**
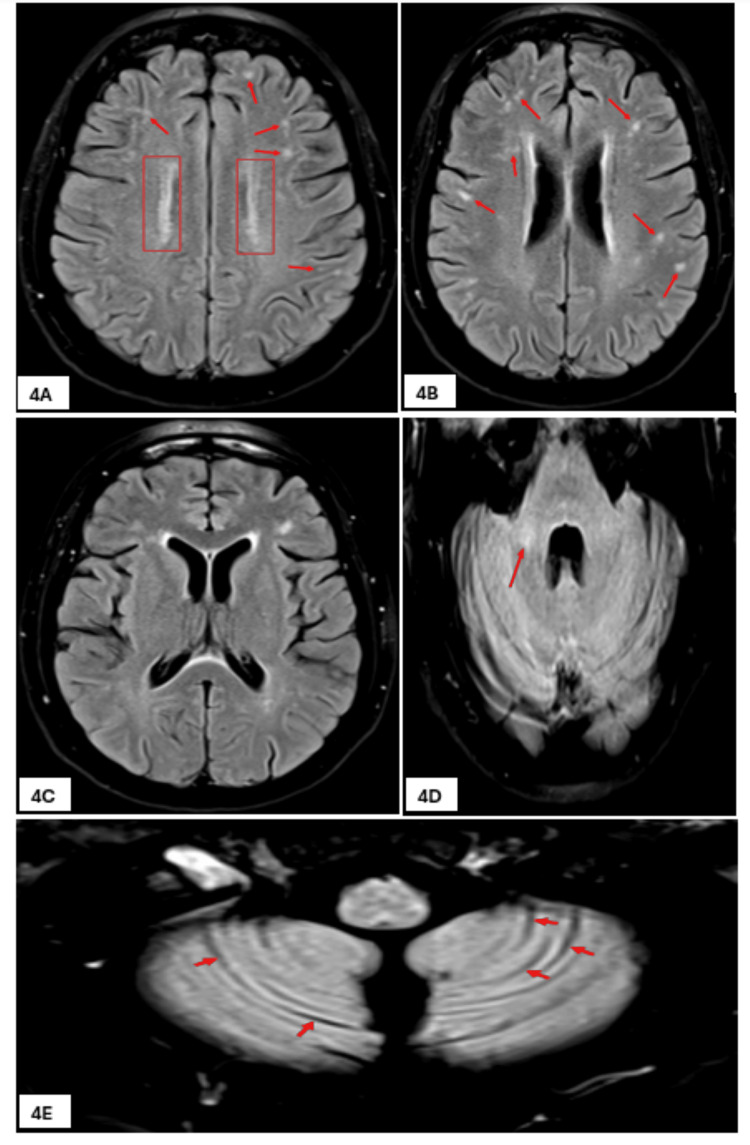
Third MRI scan of the brain. Two-dimensional axial fluid-attenuated inversion recovery (FLAIR) images showing progressive development of bilateral subcortical foci (arrows in 4A and 4B) and deep periventricular white matter sheets of FLAIR signal elevation (rectangle in 4A), with the latter showing predilection for a perimedullary venous location (4A and 4B compared with 1A, 2A, and 3A), with resolution of the basal ganglia foci (4C compared with 3B) as well as tegmental pontine signal alteration (4D dcompared with 3C), and interval development of a right middle cerebellar peduncle ill-defined focus of FLAIR signal alteration (arrow in 4D compared with 3C), and development of early cerebellar atrophic changes (arrows in 4E compared with 1D).

At the end of her two-week stay in the ICU, she was extubated and transferred to care under the neurology team for neurorehabilitation and monitoring. She was treated by a multidisciplinary team of dieticians, speech and language therapists, diabetes team, occupational therapists, and physiotherapists. Her speech and cognition improved, and clinical examination revealed bilateral proximal weakness in all four limbs with no sensory loss and symmetric action tremors, which gradually improved during inpatient stay. She was able to mobilize with the aid of a frame, and there were no significant pyramidal or extrapyramidal signs noted at this point. An ultrasound scan of the parotid glands, performed to rule out Sjogren’s syndrome as per rheumatology input, showed parotid glands that were normal in size, shape, and parenchymal echotexture. A third MRI of the brain showed newly developed focal subcortical and deep white matter high signal intensities predominantly involving the supratentorial brain with resolution of basal ganglia foci as well as tegmental pontine signal alteration (Figures 4A-4E).

This was presumed to be due to a vasculitic or inflammatory process by the neuro-radiology multidisciplinary team, especially in view of a previous pulmonary embolus. Blood testing done previously also included tests to rule out vasculitis (Table 2). Magnetic resonance angiography (MRA) was done in view of the same, which did not show any features of vasculitis. Fundoscopic examination revealed a normal fundus, visual field, visual acuity, and color vision. Repeat CSF testing showed a downward trend in mononuclear cells and the presence of GFAP IgG antibodies (Table 3). These antibodies were also found in her serum, which helped formulate the final diagnosis (Table 2). She was discharged on a weaning dose of prednisolone and continues to make good progress under the care of the community rehabilitation team. Further review has been arranged with the neurology team. A timeline of key events during her hospital and ICU stay is presented in Table 4.

**Table 4:** The patient’s timeline with key events during hospital/ICU stay. GCS: Glasgow Coma Scale; CTH: computed tomography of the head

Hospital stay	Course	ICU stay
Day 1	Admitted to the hospital - low GCS with normal CTH. Started on antimicrobials and anti-epileptics	-
Day 3	Transferred to ICU (due to persistent low GCS) 1st EEG (no evidence of epileptiform abnormalities) 1st lumbar puncture	Day 1
Day 4	Intubated	Day 2
Day 7	1st​​​​​​​ MRI (showing possible post-viral encephalitis) 2nd EEG (no epileptiform abnormalities)	Day 5
Day 11	Intravenous methylprednisolone started at 1 g/kg/day for 5 days	Day 9
Day 13	First successful sedation interruption, patient responding to verbal commands, 3rd EEG (no epileptiform abnormalities)	Day 11
Day 14	Patient is able to follow verbal commands	Day 12
Day 16	Extubated. Oral prednisolone started at 1 mg/kg/day and was weaned down at 5 mg/week	Day 14
Day 17	2nd MRI (post-infective or ischemic changes)	Day 15
Day 21	Transferred from the ICU to the Neurology unit	Day 19
Day 33	3rd MRI (new focal white matter signal abnormalities)	-
Day 40	2nd lumbar puncture, including samples for glial fibrillary acidic protein antibody testing	-
Day 41	Magnetic resonance angiogram (MRA) (unremarkable)	-
Day 48	Discharged from the hospital	-

## Discussion

Brainstem encephalitis or rhombencephalitis is a rare infectious or inflammatory condition affecting parts of the brainstem. The diagnosis can be challenging and is commonly caused by infections like Listeria [10], although there have been reports of post-viral brainstem encephalitis secondary to COVID-19 infection [11], with some cases secondary to inherited deficiency of the RNA lariat-debranching enzyme 1 (DBR1), as described by Chan et al. in their study [12].

Glial fibrillary acidic protein (GFAP) astrocytopathy is a rare autoimmune condition characterized by antibodies in the blood or cerebrospinal fluid against GFAP alpha. It is thought to be due to immune reactions involving macrophages, plasma cells, helper T cells [13], and cytotoxic T cells [4]. It is associated with viral infections like COVID-19 or malignancies like breast cancer [14,15]. There are rare instances of the coexistence of GFAP astrocytopathy and myelin oligodendrocyte glycoprotein immunoglobulin G (MOG-IgG) disease [16]. Although there are no agreed diagnostic criteria, a few typical features, such as linear perivascular radial enhancement or leptomeningeal enhancement on imaging, and the presence of IgG GFAP antibodies in the CSF, can aid in diagnosis [17]. Onset can be subacute, and the clinical course severe, with features of brain-stem involvement. Treatment primarily involves immunosuppression with steroids [11]. The long-term outcomes seem to be favorable, although relapses can be seen in 20-30% of the cases with higher rates associated with concurrent NMDAR-IgG antibodies, AQP-4 antibodies [18], and tumor at the onset of symptoms [17,18].

In the present case, the patient had multiple initial visits to the emergency department with tremors and dizziness and progressed over a course of a few weeks to develop encephalopathic features with brainstem involvement. She was started on anticonvulsive and antimicrobial therapy and extensively investigated by a multidisciplinary team, and the cause of her presentation was initially not known. An excellent response to corticosteroid therapy and MRI findings of radial perivascular enhancement indicated a possible autoimmune pathophysiology of the encephalitis [6,8]. Potential differentials included GFAP astrocytopathy, anti-NMDAR encephalitis, MOG antibody disease, and neuromyelitis optica spectrum disorder (NMOSD). Repeat CSF testing near the end of her hospital stay revealed IgG antibodies to glial fibrillary acidic protein in both the CSF and the serum. Neuronal antibodies for other autoimmune encephalitides were negative (Table 2). As illustrated in the present report, diagnosis can be challenging due to the possibility of concurrent presentation or cases mimicking CNS infections, with a subsequent risk of misdiagnosis as intracranial infections [19-22]. Potential consequences include the administration of inappropriate treatment and subsequent delays in initiating proper therapy, which may adversely affect patient outcomes. This underscores the significance of keeping a wider array of differentials and starting early empirical therapy.

After discharge, the patient experienced some initial cognitive challenges, including memory and attention difficulties, which are common in GFAP astrocytopathy. Over time and with rehabilitation, she showed significant improvement, particularly in verbal memory and problem-solving. However, mild cognitive slowing and attention issues persisted. She continues to make good progress under rehabilitation and is on a weaning dose of prednisolone. Further follow-up has been arranged.

## Conclusions

Glial fibrillary acidic protein (GFAP) astrocytopathy is a rare autoimmune condition affecting the central nervous system, characterized by the presence of immunoglobulin G antibodies against GFAP alpha in the serum or cerebrospinal fluid. It can present clinically and radiologically with features of infectious encephalitis and can pose a diagnostic challenge due to a protracted clinical course. It is important to consider autoimmune pathophysiology in cases of infectious encephalitis and to conduct relevant investigations, including CNS imaging, serological testing, and neurophysiological studies, at first instance. Empirical initiation of corticosteroid-based immunosuppression at an early stage has been suggested to reduce illness severity and improve patient outcomes, consistent with the existing literature.

## References

[1] Shan F, Long Y, Qiu W (2018). Autoimmune glial fibrillary acidic protein astrocytopathy: a review of the literature. Front Immunol.

[2] Dubey D, Pittock SJ, Kelly CR (2018). Autoimmune encephalitis epidemiology and a comparison to infectious encephalitis. Ann Neurol.

[3] Hagbohm C, Ouellette R, Flanagan EP (2024). Clinical and neuroimaging phenotypes of autoimmune glial fibrillary acidic protein astrocytopathy: a systematic review and meta-analysis. Eur J Neurol.

[4] Guo Y, Endmayr V, Zekeridou A (2024). New insights into neuropathology and pathogenesis of autoimmune glial fibrillary acidic protein meningoencephalomyelitis. Acta Neuropathol.

[5] Gravier-Dumonceau A, Ameli R, Rogemond V (2022). Glial fibrillary acidic protein autoimmunity: a French cohort study. Neurology.

[6] Kunchok A, Zekeridou A, McKeon A (2019). Autoimmune glial fibrillary acidic protein astrocytopathy. Curr Opin Neurol.

[7] Charan BD, Priya S, Goel V, Chhatarpal P, Jain S, Gupta A, Garg A (2024). Unveiling distinctive MRI characteristics in the diagnosis of GFAP astrocytopathy: a rare autoimmune neuroinflammatory disorder. Ann Indian Acad Neurol.

[8] Ma W, Huang C, Yang L, Luo J (2022). MRI findings of autoimmune glial fibrillary acidic protein astrocytopathy involving infratentorial: case report. Radiol Case Rep.

[9] Xiao J, Chen X, Shang K (2021). Clinical, neuroradiological, diagnostic and prognostic profile of autoimmune glial fibrillary acidic protein astrocytopathy: a pooled analysis of 324 cases from published data and a single-center retrospective study. J Neuroimmunol.

[10] Paranjape N (2021). Rhombencephalitis due to Listeria monocytogenes. IDCases.

[11] Shamier MC, Crijnen YS, Bogers S (2023). Brain stem encephalitis is a rare complication of COVID-19. J Neuroimmunol.

[12] Chan YH, Lundberg V, Pen JL (2024). SARS-CoV-2 brainstem encephalitis in human inherited DBR1 deficiency. J Exp Med.

[13] Shu Y, Long Y, Chang Y (2018). Brain immunohistopathology in a patient with autoimmune glial fibrillary acidic protein astrocytopathy. Neuroimmunomodulation.

[14] Cheng F, Tang K (2025). A case of COVID-19 encephalitis with anti-GFAP antibody-positive meningoencephalomyelitis. J Neurol Sci.

[15] Yaguchi T, Kimura A, Takekoshi A, Matsuo M, Tomita H, Shimohata T (2023). Autoimmune glial fibrillary acidic protein astrocytopathy associated with breast cancer: a case report. BMC Neurol.

[16] Martin AJ, Strathdee J, Wolfe N (2022). Coexistent anti-GFAP and anti-MOG antibodies presenting with isolated meningitis and papillitis: more support for overlapping pathophysiology. BMJ Neurol Open.

[17] Arzalluz-Luque J, Dumez P, Picard G (2025). Clinical course and long-term outcomes in autoimmune glial fibrillary acidic protein (GFAP) astrocytopathy. J Neurol.

[18] Gklinos P, Athanasopoulos F, Giatrakou V (2024). Unveiling GFAP astrocytopathy: insights from case studies and a comprehensive review of the literature. Antibodies (Basel).

[19] An Q, Liu L (2025). A case report of autoimmune glial fibrillary acidic protein astrocytopathy combined with Epstein-Barr virus infection. BMC Neurol.

[20] Guo Y, Guo J, Wang X (2024). Glial fibrillary acidic protein astrocytopathy presented as meningitis: a case report. Heliyon.

[21] Vu AP, Kapadia RK, Roberts JI (2025). Pearls and oy-sters: autoimmune glial fibrillary acidic protein astrocytopathy presenting as encephalomyelitis with leptomeningeal enhancement. Neurology.

[22] Bai R, An L, Du W (2025). Autoimmune glial fibrillary acidic protein astrocytopathy misdiagnosed as intracranial infectious diseases: case reports and literature review. Front Immunol.

